# Elevated Plasma BDNF in Early Primary Biliary Cholangitis: Associations with Liver Fibrosis, IL-6, IL-18, Fatigue, and Cognitive Impairment

**DOI:** 10.3390/ijms26157142

**Published:** 2025-07-24

**Authors:** Magdalena Rogalska, Sławomir Ławicki, Agnieszka Błachnio-Zabielska, Piotr Zabielski, Kamila Roszczyc-Owsiejczuk, Jacek Janica, Dagmara Bogdanowska-Charkiewicz, Aleksandra Andrzejuk, Andrzej Dąbrowski, Robert Flisiak, Paweł Rogalski

**Affiliations:** 1 Department of Infectious Diseases and Hepatology, Medical University of Białystok, 15-089 Białystok, Poland; 2 Department of Population Medicine and Lifestyle Diseases Prevention, Medical University of Białystok, 15-089 Białystok, Poland; 3 Hygiene, Epidemiology and Metabolic Disorders Department, Medical University of Białystok, 15-089 Białystok, Poland; 4 Department of Medical Biology, Medical University of Białystok, 15-089 Białystok, Poland; 5 Department of Paediatric Radiology, Medical University of Białystok, 15-089 Białystok, Poland; 6 Department of Gastroenterology and Internal Medicine, Medical University of Białystok, 15-089 Białystok, Poland; 7 Faculty of Medicine, Medical University of Białystok, 15-089 Białystok, Poland

**Keywords:** brain-derived neurotrophic factor (BDNF), cognition disorders, elastography, fatigue, fibrosis, hepatic encephalopathy, inflammation, Interleukin-6, Interleukin-18, liver, liver cirrhosis, liver function tests, primary biliary cholangitis, ultrasonography

## Abstract

Background and Aims: Primary biliary cholangitis (PBC) is a chronic autoimmune liver disease frequently associated with fatigue and mild cognitive impairment. Brain-derived neurotrophic factor (BDNF) plays key roles in neuroplasticity, immune regulation, and metabolism. This study aimed to evaluate plasma BDNF levels in early-stage PBC and examine their clinical and biochemical associations. Methods: In this observational study, plasma BDNF, IL-6, and IL-18 concentrations were measured by ELISA in 45 patients with early-stage PBC and 31 age- and sex-matched healthy controls (mean age 60.5 years; 96% women). All participants underwent liver elastography using point shear wave elastography (ElastPQ), Doppler ultrasound, laboratory testing, and assessment of cognitive function (PHES) and fatigue severity (MFIS). Non-invasive fibrosis scores (APRI, FIB-4) were calculated. Results: Median plasma BDNF concentrations were significantly higher in PBC patients than in controls [median: 21.04 ng/mL (IQR: 10.68–38.07) vs. 5.80 ng/mL (IQR: 4.58–7.54); *p* < 0.0001]. In PBC patients, higher BDNF levels correlated inversely with liver stiffness measured by ElastPQ (R = −0.39, *p* = 0.0258), spleen dimensions, splenic vein flow volume (R = −0.49, *p* = 0.0018), suggesting an association with milder liver fibrosis and early hemodynamic alterations. A trend toward association between BDNF and IL-6 levels was observed in multivariate analysis. No significant associations were found between BDNF concentrations and markers of hepatocellular injury, cognitive performance, or fatigue severity. Conclusions: Plasma BDNF concentrations are elevated in early-stage PBC and inversely correlate with liver fibrosis severity. No significant associations were found with hepatocellular injury, cognitive function, or fatigue. These findings suggest that BDNF may play a protective role against hepatic fibrogenesis, or alternatively, that BDNF concentrations may decline with advancing liver disease. Further studies are needed to clarify its significance in PBC.

## 1. Introduction

Primary biliary cholangitis (PBC) is a chronic autoimmune liver disease that predominantly affects women, with peak incidence occurring in the fifth decade of life. Although its exact etiology remains unknown, both environmental triggers and genetic predisposition are thought to contribute to its pathogenesis [1]. PBC is characterized by immune-mediated injury to the epithelial cells of the intralobular bile ducts, leading to chronic inflammation, progressive destruction of the bile ducts, and resulting cholestasis. Over time, this process may lead to liver fibrosis, cirrhosis, and ultimately liver failure. The presence of antimitochondrial antibodies, particularly the M2 subtype (AMA-M2), is a hallmark of PBC and is found in approximately 90–95% of patients [2,3].

Beyond pruritus, fatigue is the most common and disabling symptom of PBC, affecting up to 80% of individuals [4,5,6]. It is increasingly recognized as a complex condition involving both central and peripheral mechanisms, often accompanied by sleep–wake disturbances [2]. Mild cognitive impairment is also common and frequently coexists with fatigue [4,7,8,9]. Notably, both symptoms appear largely independent of liver disease stage or biochemical activity, suggesting an extrahepatic origin [10,11]. Functional imaging studies have revealed disrupted deep gray matter connectivity and impaired cerebral autoregulation, supporting the idea that PBC can affect brain function independently of liver injury [12,13,14]. These central symptoms may persist even after liver transplantation, indicating long-standing neurobiological alterations [10].

Brain-derived neurotrophic factor (BDNF) is a key neurotrophin involved in the development, survival, and plasticity of neurons. BDNF primarily exerts its effects through the tropomyosin receptor kinase B (TrkB) and can also bind to the p75 neurotrophin receptor (p75NTR) [15]. Beyond its well-established role in the central nervous system, BDNF has been increasingly recognized as a regulator of energy balance, glucose metabolism, and inflammatory responses [16,17,18].

Given its wide-ranging actions, BDNF may be relevant to several clinical aspects of PBC. Since BDNF is produced in skeletal muscle, it may contribute to peripheral mechanisms of fatigue. Its role in immune modulation and interaction with inflammatory pathways may potentially contribute to the autoimmune process and disease expression. Available data on BDNF in liver diseases remain inconclusive. Previous studies reported elevated circulating BDNF levels in non-alcoholic fatty liver disease, where they correlated with markers of hepatic injury [19]. Experimental models demonstrated increased BDNF expression in fibrotic liver tissue from patients with chronic hepatitis B and in animal models of liver fibrosis [20]. In contrast, reduced BDNF concentrations were observed in cirrhotic patients with hepatic encephalopathy, showing negative correlations with bilirubin and INR [21].

Based on these observations, we hypothesized that circulating BDNF levels are altered in patients with PBC and associated with key clinical features, including liver fibrosis, liver biochemistry, minimal hepatic encephalopathy, and fatigue severity. Given the role of systemic inflammation and prior associations of IL-6 and IL-18 with minimal hepatic encephalopathy, these cytokines were included as potential inflammatory correlates [22]. As a rare autoimmune liver disease, PBC limits large-scale studies, making detailed analyses in well-characterized cohorts especially valuable.

## 2. Results

### 2.1. Study Population

The study included 76 participants: 45 patients with primary biliary cholangitis and 31 healthy controls. The average age of the participants was 60.49 ± 6.81 years, with no statistically significant difference between groups (*p* = 0.120). Most participants were women (96%), and gender distribution did not differ significantly between groups (*p* = 1.000). Although statistically significant differences were observed in multiple laboratory parameters between PBC patients and controls, most values remained within or near the reference range. Significant differences were found in platelet count, liver enzymes (ALT, AST, GGTP, ALP), inflammatory markers (CRP, IL-6, IL-18), fibrinogen, and markers of liver fibrosis (APRI, FIB-4, ElastPQ). Elevated APRI and FIB-4 scores suggest the presence of liver fibrosis in a subset of PBC patients. A detailed comparison of laboratory results and fibrosis indicators is presented in Table 1.

### 2.2. Brain-Derived Neurotrophic Factor (BDNF) Level

**Figure 1:**
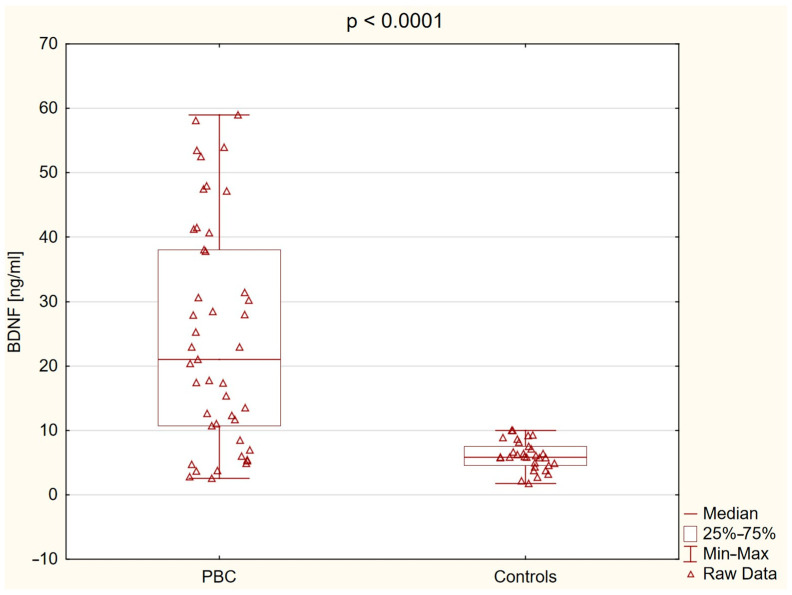
Plasma brain-derived neurotrophic factor (BDNF) levels in patients with primary biliary cholangitis (PBC) and healthy controls. Box plots demonstrate significantly elevated plasma concentrations of BDNF in the PBC group compared to the control group (*p* < 0.0001).

Plasma BDNF levels were significantly higher in patients with PBC compared to controls: 21.04 ng/mL (10.68−38.07) vs. 5.80 ng/mL (4.58−7.54), *p* < 0.0001 (Figure 1).

**Figure 2:**
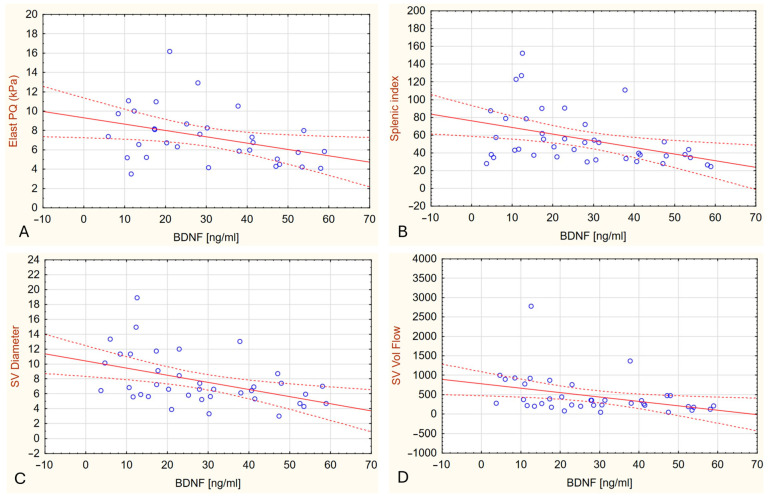
Scatter plots showing Spearman correlations between plasma BDNF levels and selected ultrasound/Doppler parameters in patients with PBC. (**A**) Liver stiffness measured by ElastPQ (R = −0.394, *p* = 0.0258). (**B**) Splenic index (R = −0.432, *p* = 0.0054). (**C**) Splenic vein diameter (SV Diameter; R = −0.443, *p* = 0.0536). (**D**) Splenic vein flow volume (SV Vol Flow; R = −0.489, *p* = 0.0018). Abbreviations: Brain-Derived Neurotrophic Factor (BDNF); Point Shear Wave Elastography (ElastPQ); Splenic Vein (SV); Volume Flow (Vol Flow).

In patients with PBC, plasma BDNF concentrations correlated negatively with liver stiffness measured by elastography (R = −0.394, *p* = 0.0258), splenic index (R = −0.432, *p* = 0.0054), splenic vein diameter (R = −0.449, *p* = 0.005), and splenic vein flow volume (R = −0.489, *p* = 0.0018) (Figure 2).

Full correlation results are presented in Supplementary Table S1. Overall, these findings suggest that higher plasma BDNF concentrations are associated with less advanced liver fibrosis and milder portal hypertension in patients with PBC.

A marked heterogeneity in plasma BDNF concentrations was observed among patients with PBC. To further investigate the clinical correlates of elevated BDNF levels, patients were stratified based on the distribution of plasma BDNF, with individuals above the third quartile (>Q3) classified as the high BDNF group and those at or below Q3 as the low BDNF group. Clinical and biochemical comparisons between these subgroups are presented in Supplementary Table S2. Patients with higher BDNF concentrations showed significantly lower liver stiffness measured by ElastPQ (*p* = 0.0020), FIB-4 scores (*p* = 0.020), and APRI scores (*p* = 0.040) compared to those with lower BDNF concentrations. No significant differences were observed for liver enzymes, inflammatory markers, cognitive performance, or fatigue severity.

### 2.3. Plasma BDNF Levels in Relation to Liver Stiffness

**Figure 3:**
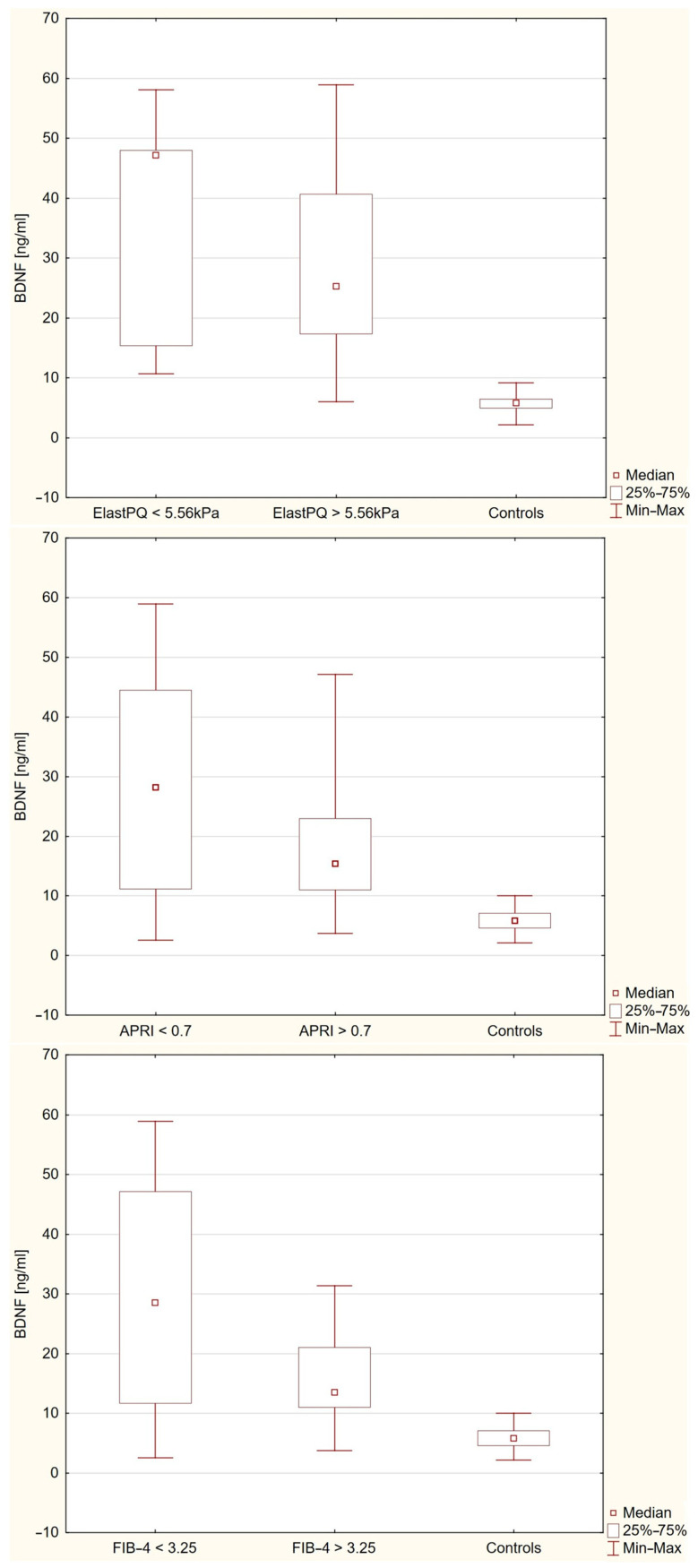
Plasma BDNF levels in patients with PBC stratified by APRI, FIB-4, and liver stiffness (ElastPQ) compared to healthy controls. Box plots show median (square), interquartile range (box), and minimum–maximum values (whiskers). Plasma BDNF concentrations were significantly higher in all PBC subgroups compared to controls (*p* < 0.0001 for APRI ≤ 0.7 and FIB-4 ≤ 3.25 vs. controls; *p* = 0.0062 and *p* = 0.0171 for APRI > 0.7 and FIB-4 > 3.25 vs. controls, respectively). No statistically significant differences were observed between PBC patients with lower versus higher fibrosis markers (*p* = 1.000 for ElastPQ; *p* = 1.000 for APRI; *p* = 0.6451 for FIB-4).

Within the PBC cohort, plasma BDNF concentrations did not differ significantly between patients with lower versus higher fibrosis markers (*p* = 1.000 for ElastPQ; *p* = 1.000 for APRI; *p* = 0.6451 for FIB-4) (Figure 3). However, plasma BDNF levels were significantly higher in all PBC subgroups compared to healthy controls (*p* < 0.0001 for APRI ≤ 0.7 and FIB-4 ≤ 3.25 vs. controls; *p* = 0.0062 and *p* = 0.0171 for APRI > 0.7 and FIB-4 > 3.25 vs. controls, respectively).

### 2.4. Association of Plasma BDNF Levels with Inflammatory and Hepatic Parameters in Multivariate Analysis

In the multiple linear regression model performed in patients with PBC and including seven potentially biologically relevant predictors (IL-6, CRP, ElastPQ, AST, ALT, GGTP, ALP), none of the variables reached statistical significance as an independent predictor of plasma BDNF concentration. Among individual predictors, IL-6 demonstrated a trend towards association with BDNF levels (β = 0.40, *p* = 0.058). The overall model explained 49.6% of the variance in BDNF levels (R^2^ = 0.496) but did not achieve statistical significance (F [7,16] = 2.25, *p* = 0.085).

### 2.5. Association Between BDNF Levels, Cognitive Performance, and Fatigue

Minimal hepatic encephalopathy (MHE), defined as a PHES score below –4, was identified in 8 out of 44 PBC patients (18.2%) and in none of the healthy controls (0%), with a statistically significant difference between groups (*p* = 0.02). One patient with an MMSE score below the normal range was excluded from further neuropsychological testing. Overall cognitive performance, as assessed by PHES and its individual components, was significantly impaired in the PBC group. The median PHES score was −1.00 (IQR: –3.00 to 0.50) in PBC patients versus 2.00 (IQR: 1.00 to 3.00) in controls (*p* < 0.0001). Significant differences were also observed for NCT-A, NCT-B, SDT, and DST (*p* < 0.0001 for all except DST, *p* = 0.02) (see Table 2).

**Figure 4:**
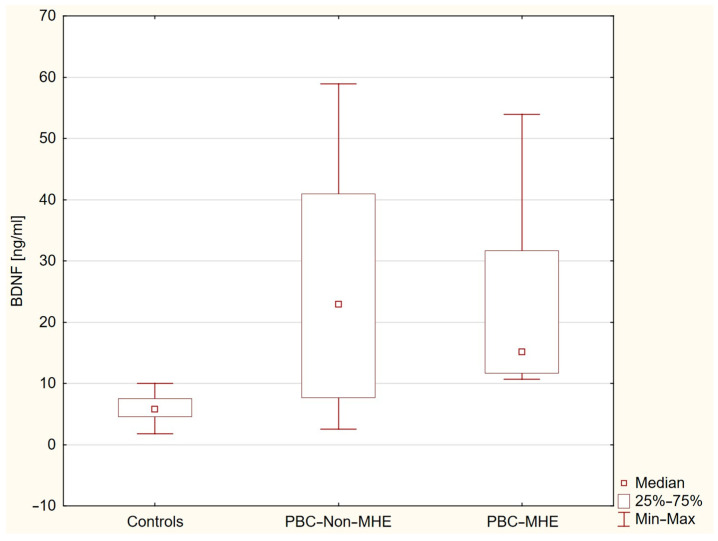
Plasma BDNF concentrations in healthy controls and PBC patients stratified by minimal hepatic encephalopathy (MHE) status. Box plots illustrate significantly elevated BDNF levels in both PBC groups (MHE and non-MHE) compared to controls (*p* < 0.0001 and *p* = 0.0015, respectively). No significant difference was found between patients with and without MHE (*p* = 1.0000).

Although plasma BDNF levels differed significantly among groups stratified by MHE status (*p* < 0.0001), post hoc comparisons revealed no significant difference between PBC patients with and without MHE (*p* = 1.0000). Median BDNF concentrations were lowest in healthy controls [5.80 ng/mL (IQR: 4.58–7.54); *n* = 31], higher in PBC patients without MHE [22.94 ng/mL (IQR: 7.69–40.95); *n* = 36], and intermediate in those with MHE [15.17 ng/mL (IQR: 11.66–31.65); *n* = 8]. Both PBC subgroups showed significantly elevated BDNF levels compared to controls (*p* < 0.0001 and *p* = 0.0015, respectively), suggesting that increased BDNF is a general feature of early PBC, independent of MHE status (Figure 4).

In the multivariate logistic regression model predicting cognitive impairment (PHES < 0), none of the included variables reached statistical significance. Notably, plasma BDNF concentration was not independently associated with the outcome (*p* = 0.943). Similarly, no significant associations were found for age (*p* = 0.889), liver stiffness assessed by ElastPQ (*p* = 0.752), serum ammonia levels (*p* = 0.664), ALT (*p* = 0.477), AST (*p* = 0.407), education years (*p* = 0.914), or IL-6 concentration (*p* = 0.414).

Fatigue severity was significantly greater in PBC patients compared to controls. The median total MFIS score was 31.0 (IQR: 15.0–45.0) in the PBC group versus 17.5 (IQR: 14.5–22.5) in controls (*p* = 0.04). In addition to total MFIS scores, subscale analysis revealed that physical fatigue scores were higher in the PBC group (median: 13.0, IQR: 7.0–19.0) compared to controls (median 8.5, IQR: 7.0–12.5; *p* = 0,05). Similar trends were observed for the psychosocial and cognitive subscales (*p* = 0.08 and *p* = 0.075, respectively; Table 3). No significant correlations were found between plasma BDNF concentrations and cognitive performance (PHES, MMSE) or fatigue severity (MFIS) in PBC patients.

## 3. Discussion

To the best of our knowledge, this study is the first to demonstrate significantly elevated plasma BDNF levels in patients with early-stage primary biliary cholangitis compared to healthy controls. Although BDNF is primarily recognized for its role in the central nervous system (CNS), it also exerts pleiotropic effects in peripheral tissues, including regulation of immune responses, energy homeostasis, and vascular remodeling [15,23]. Beyond neuronal sources, BDNF is produced by hepatocytes, skeletal muscle cells, and vascular endothelial cells. Physical exercise has been shown to stimulate BDNF release from skeletal muscle, and recent studies suggest that muscle-derived BDNF contributes to energy regulation [17,18,24,25]. These peripheral sources and physiological roles of BDNF may help explain the elevated circulating levels observed in PBC.

Our findings demonstrate that plasma BDNF concentrations were significantly elevated in patients with PBC compared to healthy controls. However, no significant positive correlations were observed between BDNF levels and markers of hepatocellular injury or cholestasis. Instead, BDNF concentrations showed negative correlations with liver stiffness assessed by elastography and several portal hemodynamic parameters, suggesting an inverse relationship with the severity of hepatic remodeling. These results indicate that while increased BDNF is a feature of PBC, it may not directly reflect the extent of hepatocellular injury. Similar elevations in circulating BDNF have been reported in non-alcoholic fatty liver disease (NAFLD); however, in contrast to our findings, Hattori et al. demonstrated a positive association between BDNF levels and hepatic injury markers in NAFLD [19].

Another potential contributor to elevated plasma BDNF concentrations in early-stage PBC is platelet activation [26]. Platelets are a major peripheral source of BDNF, storing the protein predominantly in α-granules and releasing it upon activation by agonists such as thrombin, adenosine diphosphate (ADP), or serotonin. This process involves classical platelet receptors (e.g., protease-activated receptor 1 [PAR-1]) and may also engage TrkB-mediated intracellular signaling pathways, including phosphoinositide 3-kinase (PI3K) and protein kinase C (PKC) [27]. In our earlier work, we reported elevated plasma levels of soluble glycoprotein V (sGPV), a marker of thrombin-induced platelet activation, in patients with early-stage PBC compared to healthy controls [28]. However, sGPV reflects proteolytic cleavage associated with thrombin signaling and does not directly represent the α-granule secretion pathway responsible for BDNF release. Therefore, it remains unclear whether the observed increase in plasma BDNF is driven predominantly by thrombin-mediated activation or involves additional mechanisms.

Although plasma BDNF concentrations were elevated in patients with PBC, within the patient cohort higher BDNF levels were associated with lower liver stiffness and lower non-invasive fibrosis scores. Experimental findings by Sun et al. demonstrated upregulation of BDNF, glial fibrillary acidic protein (GFAP), and growth-associated protein 43 (GAP43) in fibrotic liver tissues from patients with chronic hepatitis B and in mouse models of fibrosis [20]. In vitro, BDNF stimulation of hepatic stellate cells increased the expression of pro-inflammatory cytokines and fibrogenic markers. These experimental observations imply that BDNF may participate in hepatic fibrogenesis under certain conditions; however, our clinical data suggest that elevated circulating BDNF in PBC is not directly linked to more advanced fibrosis. However, our findings raise the possibility that circulating BDNF may serve as a non-invasive biomarker for milder liver fibrosis in early-stage PBC. Although causality cannot be established, the consistent inverse associations with liver stiffness, APRI, and FIB-4 suggest that higher BDNF levels may reflect a favorable fibrotic profile. Validation in longitudinal studies is needed to assess its predictive utility.

Plasma BDNF concentrations did not significantly correlate with systemic inflammatory markers; however, a trend toward association with IL-6 was identified in the multivariate model, which may reflect the complex neuroimmune regulation of BDNF in chronic inflammatory states. This finding may suggest a potential link between systemic inflammation and BDNF regulation in early-stage PBC. Similar associations have been reported in other inflammatory conditions, where elevated cytokine levels, including IL-6 and IL-18, have been linked to altered BDNF expression [29,30]. In the context of PBC, the autoimmune nature of the disease—characterized by the presence of antimitochondrial antibodies and autoreactive lymphocyte activation—may amplify inflammatory pathways that influence BDNF signaling. Although the direct impact of AMA on BDNF expression has not been investigated, persistent immune activation likely contributes to altered neuroimmune interactions in this population. Furthermore, systemic inflammation may impact the central nervous system by increasing blood–brain barrier permeability, activating glial cells, and disrupting cerebral homeostasis [31,32]. In PBC, where cognitive impairment and fatigue are prevalent, such mechanisms, together with potential alterations in BDNF signaling within the CNS, may contribute to neurocognitive symptoms, although this relationship remains to be clarified.

Minimal hepatic encephalopathy was more prevalent among PBC patients than healthy controls; however, plasma BDNF levels did not differ significantly between PBC patients with and without MHE, indicating that elevated BDNF is not specific to the presence of MHE in early-stage PBC. BDNF concentrations were significantly higher in both PBC subgroups (with and without MHE) compared to controls. No significant correlations were observed between plasma BDNF levels and cognitive test performance, and BDNF was not independently associated with cognition in multivariate analysis. These findings suggest that increased BDNF may be a general feature of early-stage PBC rather than being directly related to cognitive impairment. Our findings in early-stage PBC patients without cirrhosis contrast with limited observations in cirrhotic populations, where reduced serum BDNF levels have been reported in patients with hepatic encephalopathy. In a study by Stawicka et al., BDNF concentrations were significantly lower in individuals with MHE and correlated negatively with bilirubin and INR, and positively with platelet count [21]. Additionally, patients with sleep disturbances showed reduced BDNF levels. Experimental studies in rodent models of hepatic encephalopathy have demonstrated increased cerebellar BDNF expression associated with neuroinflammatory changes and motor dysfunction [33,34,35,36]. Specifically, Arenas et al. showed that microglia-derived BDNF activated the TrkB–PI3K–AKT–NF-κB signaling pathway in Purkinje neurons, leading to elevated expression of TNFα, HMGB1, and glutaminase I, ultimately contributing to neuronal dysfunction [36]. Enhanced BDNF–TrkB signaling was also linked to potentiation of cerebellar GABAergic neurotransmission and motor impairment [34]. Although our study did not assess central mechanisms directly and no correlations between plasma BDNF and cognitive performance were found, the elevation of BDNF in PBC may still reflect early neuroimmune changes potentially relevant to cognitive symptoms. Further research is needed to clarify the role of BDNF in neuropsychiatric manifestations of PBC. While peripheral BDNF likely reflects systemic influences, its relationship to central nervous system changes remains uncertain and warrants investigation using direct neurobiological measures.

Experimental evidence suggests that BDNF may influence peripheral energy regulation and mitochondrial function. Impaired muscle mitochondrial efficiency and delayed recovery from exertion have been reported in patients with PBC, supporting the presence of a peripheral component to fatigue in this population [11]. Moreover, studies indicate that BDNF plays a critical role in maintaining mitochondrial quality control in skeletal muscle, and that altered BDNF signaling can impair mitochondrial efficiency and metabolism, potentially contributing to fatigue [17]. Further research is needed to explore the potential links between BDNF dysregulation and fatigue in PBC. Nevertheless, our findings do not support a direct association between plasma BDNF levels and fatigue severity in early-stage PBC.

This study has several limitations. A key limitation of our study is the relatively small sample size, comprising 45 patients with early-stage PBC and 31 healthy controls. While significant differences in BDNF levels and associations with clinical parameters were observed, the modest cohort size may limit statistical power, particularly in multivariate analyses, and restrict the generalizability of our findings. Larger, multicenter studies are needed to validate these results and explore subgroup-specific patterns. Second, the cross-sectional design of the study precludes the assessment of temporal or causal relationships. It remains unclear whether elevated plasma BDNF levels represent an early compensatory response, a protective factor, or a consequence of milder disease severity. Longitudinal studies with repeated BDNF measurements and follow-up of fibrosis progression or symptom evolution would be instrumental in elucidating the dynamic role of BDNF in the course of PBC. Third, BDNF concentrations were measured only in plasma, which may not fully reflect central nervous system activity. Finally, all patients in our cohort were on stable UDCA therapy, which may have influenced systemic inflammatory or metabolic parameters. Although there is currently limited evidence that UDCA directly modulates BDNF expression, we cannot exclude a potential confounding effect. Future studies including UDCA-naïve patients or examining BDNF dynamics pre- and post-treatment would help clarify this aspect.

## 4. Methods and Materials

This was a single-center, observational study including adult patients with a confirmed diagnosis of PBC. Patients were recruited during routine outpatient visits. All participants were in stable clinical condition and managed on an outpatient basis. A history of hepatic decompensation—defined as prior ascites, overt hepatic encephalopathy, or variceal bleeding–was an exclusion criterion. The diagnosis of PBC was based on standard clinical criteria, including elevated cholestatic liver enzymes and the presence of AMA or other disease-specific autoantibodies [37]. All patients were receiving treatment with ursodeoxycholic acid (UDCA) at the recommended dose (13–15 mg/kg/day) [37]. The control group consisted predominantly of age- and sex-matched healthcare workers without evidence of liver disease. All participants underwent a standardized clinical evaluation, including medical history assessment, physical examination, and basic laboratory tests. Blood samples were collected, centrifuged, and plasma was frozen and stored at −85 °C until further analysis. ELISA-based assays were used to determine BDNF, IL-6, and IL-18 levels. Liver ultrasound with Doppler, elastography, as well as psychological and fatigue assessments, were performed as detailed below.

### 4.1. Brain-Derived Neurotrophic Factor Measurement

Plasma levels of BDNF were measured using the DuoSet ELISA Development Kit for Human/Mouse BDNF (R&D Systems, Cat. No. DY248, Minneapolis, MN, USA), following the manufacturer’s instructions. High-binding 96-well plates were coated overnight at 4 °C with the capture antibody. After blocking, plasma samples and BDNF standards were added and incubated for 2 h at room temperature. Detection antibody and streptavidin-HRP were applied in subsequent steps, followed by substrate solution. The reaction was stopped with stop solution, and absorbance was read at 450 nm with correction at 570 nm. All samples were analyzed in duplicate, and BDNF concentrations were calculated using a standard curve.

### 4.2. Interleukin-6 (Il-6) Measurement

Serum levels of interleukin-6 (IL-6) were measured using a Human IL-6 ELISA Kit (RAB0306, Sigma-Aldrich, St. Louis, MO, USA), based on the sandwich ELISA method. The assay had a detection range of 1.37–1000 pg/mL and a sensitivity below 3 pg/mL. Samples were prepared according to the manufacturer’s protocol and incubated with biotinylated detection antibody and HRP-conjugated streptavidin. Absorbance was measured at 450 nm. The assay demonstrated recovery rates of 92–96%, with intra-assay and inter-assay coefficients of variation below 10% and 12%, respectively.

### 4.3. Interleukin-18 (IL-18) Measurement

Serum IL-18 concentrations were measured using a commercial ELISA kit (Human IL-18 ELISA Kit, Thermo Fisher Scientific, Catalog No. BMS267-2, Waltham, MA, USA) following the manufacturer’s protocol. Absorbance was read at 450 nm, and IL-18 levels were calculated from a standard curve (78–5000 pg/mL). The assay sensitivity was 9 pg/mL.

### 4.4. Liver Ultrasound and Elastography

Liver stiffness was measured using point shear wave elastography (ElastPQ, Philips Healthcare, Bothell, WA, USA) with a C5-1 convex probe on an iU22 ultrasound system. Ten valid ElastPQ measurements were obtained from the right liver lobe, and the median value was used for analysis. Results were expressed in kilopascals (kPa). Measurements with an interquartile range to median ratio (IQR/M) greater than 30% were considered unreliable and repeated. A liver stiffness value above 5.56 kPa was defined as indicative of significant fibrosis [38]. Doppler ultrasound was also performed to assess portal vein diameter, flow velocity, and the presence of collateral circulation or signs of portal hypertension. Standard liver ultrasound measurements were included.

### 4.5. Non-Invasive Markers of Liver Fibrosis

Liver fibrosis was additionally assessed using the AST to Platelet Ratio Index (APRI) and the Fibrosis-4 (FIB-4) score [39,40]. Both indices were calculated using standard formulas based on routine laboratory parameters. An APRI score above 0.7 and a FIB-4 score above 3.25 were considered indicative of advanced liver fibrosis.

### 4.6. Neuropsychological and Fatigue Assessment

All psychological assessments were performed by a trained psychologist in a quiet, distraction-free setting. The Mini-Mental State Examination (MMSE) was used to exclude overt cognitive impairment. Minimal hepatic encephalopathy (MHE) was assessed using the Portosystemic Encephalopathy (PSE) Syndrome Test, which includes five paper-and-pencil tasks (NCT-A, NCT-B, DST, SDT, and LTT) evaluating attention, psychomotor speed, and visuospatial skills. The Psychometric Hepatic Encephalopathy Score (PHES) was calculated based on age-adjusted norms [41]. A PHES score below −4 indicated the presence of MHE.

To increase sensitivity for detecting milder cognitive impairment, cognitive dysfunction was defined as PHES < 0 rather than the conventional diagnostic threshold for MHE. This approach was based on previous observations that many patients with PBC report cognitive complaints or show subtle psychometric decline without meeting formal criteria for MHE and is consistent with studies emphasizing a continuum of cognitive dysfunction in chronic liver disease. To increase sensitivity for detecting milder cognitive impairment, cognitive dysfunction was defined as PHES < 0 rather than the conventional diagnostic threshold of PHES < –4 for MHE. This threshold is supported by previous studies suggesting that subtle cognitive decline may occur in patients without overt MHE and better reflects the continuum of cognitive symptoms in early-stage PBC [7].

Fatigue was measured with the Modified Fatigue Impact Scale (MFIS), a 21-item self-reported questionnaire assessing the impact of fatigue on physical, cognitive, and psychosocial functioning [42]. Each item is rated from 0 (never) to 4 (almost always), with a total score range of 0–84. Higher scores reflect more severe fatigue.

### 4.7. Statistical Analysis

Continuous variables are presented as mean ± standard deviation (SD) or median with interquartile range (IQR: 25th–75th percentile), depending on distribution assessed by the Shapiro–Wilk test. Categorical variables are expressed as counts and percentages. Group comparisons were performed using the chi-square test or Fisher’s exact test for categorical variables. For continuous variables, Student’s *t*-test was used when data followed a normal distribution, and the Mann–Whitney U test was applied otherwise. Associations between continuous variables were assessed using Spearman’s rank correlation coefficient. Differences in plasma BDNF levels across multiple groups were analyzed using the Kruskal–Wallis test with post hoc Bonferroni correction. A two-tailed *p*-value <0.05 was considered statistically significant.

To identify independent predictors of plasma BDNF concentration, a multiple linear regression model was constructed including IL-6, CRP, liver stiffness (ElastPQ), and selected liver enzymes (AST, ALT, GGTP, ALP). Variables were entered simultaneously using the enter method. Cases with missing data were excluded using casewise deletion. No substantial collinearity was observed between predictors. Statistical significance was defined as *p* < 0.05.

To identify factors independently associated with cognitive dysfunction (defined as PHES < 0), univariate logistic regression analyses were first conducted for candidate predictors, including age, liver stiffness (ElastPQ, kPa), plasma BDNF (ng/mL), ALT, AST, ammonia, IL-6, and years of education. Variables with *p* ≤ 0.10 in univariate analysis were included in the multivariate logistic regression model. All predictors were analyzed as continuous variables without transformation. Variables were entered simultaneously using the enter method. Results are reported as odds ratios (OR) with 95% confidence intervals (CI) and Wald statistics.

All analyses were performed using Data Science Workbench, version 14 (Cloud Software Group, Inc., 2023, Fort Lauderdale, FL, USA).

### 4.8. Ethical Considerations

The study was conducted in accordance with the principles of the Declaration of Helsinki. Written informed consent was obtained from all participants prior to inclusion. The study protocol was approved by the Institutional Bioethics Committee (APK.002.108.2023).

## 5. Conclusions

In summary, our findings demonstrate that plasma BDNF concentrations are significantly elevated in patients with early-stage PBC compared to healthy controls. Elevated BDNF levels were not associated with markers of hepatocellular injury, cognitive impairment, or fatigue severity. Higher BDNF concentrations correlated with lower liver stiffness and fibrosis scores, suggesting a potential protective role against hepatic fibrogenesis. Alternatively, these results may reflect a decline in BDNF with progression of chronic liver disease. Further studies are needed to better understand the role of BDNF in the pathophysiology of PBC.

## Figures and Tables

**Table 1:** Comparison of controls and the PBC group.

Variable	Controls (*n* = 31)	PBC Group (*n* = 45)	*p*
Age (years)	59.06 ± 5.42	61.51 ± 7.41	0.120
Gender (F/%)	30 (96.8%)	43 (95.6%)	1.000
WBC (×10^3^/µL)	6.22 ± 1.65	5.55 ± 1.88	0.137
RBC (×10^6^/µL)	4.49 (4.27–4.82)	4.37 (4.04–4.57)	0.104
HGB (g/dL)	13.55 (12.90–14.20)	12.9 (12.10–14.00)	0.080
PLT (×10^3^/µL)	243.92 ± 61.96	185.42 ± 82.22	0.003
ALT (IU/L)	17.0 (15.00–21.00)	28.0 (22.00–45.00)	<0.0001
AST (IU/L)	19.0 (16.00–24.00)	32.5 (26.00–43.00)	<0.0001
GGTP (IU/L)	19.0 (16.5–23.0)	68.0 (43.0–96.0)	0.0001
ALP (IU/L)	62.0 (53.0–71.0)	123.5 (93–173)	<0.0001
Ammonia (µmol/L)	30.2 (23.8–33.9)	36.25 (29.6–47.8)	0.045
Bilirubin (mg/dL)	0.63 (0.53–0.72)	0.81 (0.59–1.31)	0.024
Creatinine (mg/dL)	0.71 (0.63–0.82)	0.76 (0.69–0.92)	0.175
PT (s)	13.35 (12.70–13.75)	12.55 (11.90–13.10)	0.015
Fibrinogen (mg/dL)	341.46 ± 52.46	401.67 ± 75.65	0.001
CRP (mg/L)	1.1 (1.00–2.10)	4.92 (2.30–7.80)	<0.0001
IL-6 (pg/mL)	233.6 (152.2–315.2)	344.9 (252.0–573.4)	0.0003
IL-18 (pg/mL)	213.60 (190.0–270.8)	283.60 (236.0–358.6)	0.0052
APRI	0.22 (0.17–0.24)	0.45 (0.27–0.87)	<0.0001
FIB-4	1.23 (1.02–1.30)	1.79 (1.40–3.57)	<0.0001
ElastPQ (kPa)	3.26 (2.89–3.85)	6.74 (5.18–8.46)	<0.0001

Abbreviations: Alanine Aminotransferase (ALT), Alkaline Phosphatase (ALP), Ammonia (NH_3_), Aspartate Aminotransferase (AST), Aspartate Aminotransferase-to-Platelet Ratio Index (APRI), C-Reactive Protein (CRP), Fibrosis-4 Index (FIB-4), Gamma-Glutamyl Transpeptidase (GGTP), Hemoglobin (HGB), Interleukin-1 Beta (IL-1B), Interleukin-6 (IL-6), Interleukin-18 (IL-18), Platelet Count (PLT), Primary Biliary Cholangitis (PBC), Prothrombin Time (PT), Red Blood Cell Count (RBC), White Blood Cell Count (WBC), Liver Stiffness by ElastPQ (ElastPQ).

**Table 2:** Results of the Psychometric Hepatic Encephalopathy Score (PHES) and its components in PBC patients and controls.

Variable	Controls	PBC Group	*p*
PHES	2.00 (1.00–3.00)	–1.00 (–3.00–0.50)	<0.0001
NCT-A	1.00 (0.00–1.00)	0.00 (–1.00–0.00)	<0.0001
NCT-B	1.00 (0.00–1.00)	0.00 (–1.00–0.00)	<0.0001
SDT	0.00 (0.00–1.00)	0.00 (–1.50–0.00)	<0.0001
DST	0.00 (0.00–0.00)	0.00 (–1.00–0.00)	0.02
LTT mistakes	0.00 (0.00–1.00)	0.00 (0.00–0.50)	0.28
LTT time	0.00 (0.00–1.00)	0.00 (0.00–0.00)	0.39

Data are presented as median (25th–75th percentile). Differences between groups were assessed using the Mann–Whitney U test. Abbreviations: PHES—Psychometric Hepatic Encephalopathy Score; NCT-A—Number Connection Test A; NCT-B—Number Connection Test B; SDT—Serial Dotting Test; DST—Digit Symbol Test; LTT—Line Tracing Test.

**Table 3:** Fatigue scores (MFIS) in PBC patients and controls.

Variable	Controls (*n* = 28)	PBC Group (*n* = 27)	*p*
MFIS—total	17.5 (14.5–22.5)	31.0 (15.0–45.0)	0.04
MFIS_f	8.5 (7.0–12.5)	13.0 (7.0–19.0)	0.05
MFIS_p	6.0 (4.5–12.0)	12.0 (4.0–18.0)	0.08
MFIS_s	2.32 ± 1.66	3.41 ± 2.63	0.075

Data are presented as median (25th–75th percentile), except for MFIS_s, which is presented as mean ± standard deviation. Differences between groups were assessed using the Mann–Whitney U test (for MFIS total, f, and p) and Welch’s *t*-test (for MFIS_s). Abbreviations: MFIS—Modified Fatigue Impact Scale; MFIS_f—physical subscale; MFIS_p—psychosocial subscale; MFIS_s—cognitive subscale.

## Data Availability

The raw data supporting the conclusions of this article will be made available by the authors on request.

## Supplementary Materials

The following supporting information can be downloaded at: https://www.mdpi.com/article/10.3390/ijms26157142/s1.
