# Supplementary material for: Elevated Plasma BDNF in Early Primary Biliary Cholangitis: Associations with Liver Fibrosis, IL-6, IL-18, Fatigue, and Cognitive Impairment
Source: Int J Mol Sci. 2025 Jul 24;26(15):7142. doi: 10.3390/ijms26157142 (PMC12346204; doi:10.3390/ijms26157142)
Supplement: Supplementary file 1 [file ijms-26-07142-s001.zip › ijms-3758051-supplementary.pdf]

Supplementary Table S1. Correlations between serum BDNF levels [ng/ml] and clinical or biochemical parameters.

Pair of Variables	Spearman (R)	t(N-2)	p-value
BDNF [ng/mL] & Elast PQ (kPa)	–0.393695	–2.3458	0.0258
BDNF [ng/mL] & Spleen Length	–0.382154	–2.5493	0.0150
BDNF [ng/mL] & Spleen width	–0.398593	–2.6797	0.0108
BDNF [ng/mL] & Spleen cross-sectional area	–0.333881	–2.1844	0.0353
BDNF [ng/mL] & Splenic index	–0.432197	–2.9774	0.0054
BDNF [ng/mL] & HA TAM	–0.555081	–3.8335	0.0005
BDNF [ng/mL] & SPI	–0.390374	–2.5085	0.0169
BDNF [ng/mL] & SMV diameter (mm)	–0.359039	–2.3081	0.0268
BDNF [ng/mL] & SMV Area	–0.364412	–2.3479	0.0245
BDNF [ng/mL] & SV Diameter	–0.442827	–2.9634	0.0054
BDNF [ng/mL] & SV Vol Flow	–0.489193	–3.3653	0.0018
BDNF [ng/mL] & SV Area	–0.446597	–2.9948	0.0049

Abbreviations: Brain-Derived Neurotrophic Factor (BDNF), Elastography Point Quantification (ElastPQ), Hepatic Artery Time-Averaged Maximum Velocity (HA TAM), Splenic Index (SPI), Superior Mesenteric Vein (SMV), Splenic Vein (SV).

Supplementary Table S2. Comparison of clinical and biochemical parameters between patients with low (<Q3) and high (>Q3) plasma BDNF concentrations

Variable	Low BDNF group	High BDNF group	p-value
ElastPQ (kPa)	8.23 ± 3.03	5.61 ± 1.32	0.0020
FIB-4	2.35 (1.61–4.07)	1.53 (1.32–1.74)	0.020
APRI	0.56 (0.30–1.41)	0.28 (0.23–0.54)	0.040
ALP (IU/L)	129 (98–184)	97 (65–141)	0.120
GGTP (IU/L)	77 (35–107)	52 (43–79)	0.300
ALT (IU/L)	31 (24–56)	27 (21–42)	0.180
AST (IU/L)	33 (26–57)	30 (22–35)	0.220
IL-6 (pg/mL)	334 (252–497)	385 (245–1030)	0.320
IL-18 (pg/mL)	272 (233–309)	357 (244–438)	0.200
CRP (mg/L)	5.0 (1.8–8.3)	4.7 (3.3–7.5)	0.980
PHES	–1.97 ± 3.69	–0.91 ± 2.30	0.3765
MFIS_total	31.23 ± 18.98	24.80 ± 12.79	0.4809

Values are expressed as mean ± standard deviation (SD) or median (interquartile range, IQR), as appropriate. p-values were calculated using Welch's t-test for normally distributed variables (ElastPQ, PHES, MFIS_total) and Mann–Whitney U test for non-normally distributed variables. Abbreviations: Alanine Aminotransferase (ALT), Alkaline Phosphatase (ALP), Aspartate Aminotransferase (AST), Gamma-Glutamyl Transferase (GGTP), C-Reactive Protein (CRP), Interleukin-6 (IL-6), Interleukin-18 (IL-18), Psychometric Hepatic Encephalopathy Score (PHES), Modified Fatigue Impact Scale (MFIS), Point Shear Wave Elastography (ElastPQ).
